# Exploring Dog and Cat Management Practices in Multispecies Households and Their Association with the Pet-Owner Relationship

**DOI:** 10.3390/ani14233465

**Published:** 2024-11-30

**Authors:** Laura Menchetti, Giacomo Riggio, Silvia Calipari, Chiara Mariti, David J. Menor-Campos, Silvana Diverio

**Affiliations:** 1 School of Biosciences and Veterinary Medicine, Camerino University, Via Circonvallazione 93/95, 62024 Matelica, Italy; laura.menchetti@unicam.it; 2 Laboratory of Ethology and Animal Welfare (LEBA), Department of Veterinary Medicine, University of Perugia, Via San Costanzo 4, 06126 Perugia, Italy; giacomoriggio@gmail.com (G.R.); schatzi86@gmail.com (S.C.); 3 Department of Veterinary Sciences, University of Pisa, Viale delle Piagge 2, 56124 Pisa, Italy; chiara.mariti@unipi.it; 4 Department of Medicine and Animal Surgery, University of Cordoba, Rabanales University Campus, Ctra, Madrid-Cádiz, km 385, 14014 Córdoba, Spain; pv2mecad@uco.es

**Keywords:** animal husbandry, human–animal relationship, multipet household, companion animals, animal welfare, attachment, human–animal interactions

## Abstract

This study aimed to investigate differences and similarities of the pet–owner relationship and management of dogs and cats living in the same household. An online survey was completed by 1270 individuals who owned both dog(s) and cat(s). Most of the respondents owned only one dog (56.4%) but two or more cats (63.1%; *p* < 0.001). Moreover, cats were acquired earlier and were more likely to be neutered than dogs (*p* < 0.001). The neutering rate was also affected by breed (*p* < 0.001), age (*p* < 0.05), and the number of pets owned (*p* < 0.05). Two types of owners were identified according to how they managed their pets: owners who keep cats both indoors and outdoors and dogs indoors (65.3%) and owners who keep cats indoors and dogs outdoors (34.7%). The former group was characterized by a higher percentage of owners younger than 25 years (*p* < 0.01), owners with more than one dog or cat, large dogs (*p* < 0.001), and intact (*p* < 0.04) and mix-breed cats (*p* < 0.001). Finally, most respondents described the relationship with their cats (93.0%) and dogs (97.0%) as “loving” or “friendly”. Keeping cats indoors (*p* < 0.05) and allowing dogs and cats to sleep in the owners’ bedroom were associated with a stronger perceived pet–owner bond. Implications for cat and dog welfare are discussed.

## Simple Summary

Dogs and cats are the most popular pets, and many of them share the same living environment. However, very few studies have focused on multispecies households. This study aimed at investigating whether pet management practices by multispecies owners may differ depending on the species. Furthermore, we aimed to assess whether specific pet management practices may be associated with the quality of the affective bond towards the animal. More than 1200 multispecies owners completed our questionnaire. The results suggest that many management aspects differ between cats and dogs, including their age at acquisition and their age at neutering, both of which are lower for cats. Regardless of the species, two major management strategies could be identified: dogs kept outdoors and cats kept indoors, and dogs kept indoors and cats kept both indoors and outdoors. These types of management strategies primarily depended on the owner’s age, the number of animals owned, and the size of the dog, as well as the sexual status and breed of the cat. Finally, owners who kept their cats indoors and allowed both their dogs and cats to sleep in their bedroom reported a stronger perceived bond with their animals.

## 1. Introduction

In Europe, dogs and cats are the most popular pets, so much so that approximately a quarter of the population shares its living environment with at least one of them [1]. In Italy, a recent survey reported that the total number of family dogs and cats is around 19 mln, with 10.2 mln felines and 8.8 mln canines [2].

Dogs are traditionally considered “man’s best friend” [3,4] and, throughout their coexistence with humans, have been used in multiple working and social contexts [5]. On the other hand, cats have been historically valued as a natural means of rodent control, although their primary role in human society has progressively shifted to that of companion animal [6], to the point of becoming the most popular pet in some European countries [1]. Although both domestic dogs and cats are often considered family members [7,8], people perceive marked species-specific differences in their personalities and behavioral traits [9,10]. For instance, cats are often described as more independent, less sociable, and easier to care for compared to dogs [11]. Contrastingly, dogs are perceived as more financially and physically demanding but also more sociable and more emotionally supportive [12].

Previous findings suggest that such species-specific peculiarities in people’s perceptions of dog and cat behavioral profiles and needs may affect several dimensions of their husbandry and management (e.g., the urban features of the area where the animal lives [13,14], the provision of outdoor access [13,15,16,17], the structure of the adopting human family [13], the frequency and type of interactions [12,18,19], the amount of time spent in social isolation [15,17], neutering [15]), with consequent implications for their welfare [20,21,22,23,24].

While dog and cat owners’ management practices and the factors that may affect them have been largely investigated in single-species households, there is very little knowledge of how these animals are managed in multispecies households. Most studies targeting multispecies households mainly focused on the relationship/interactions between non-human animals [25,26,27] rather than husbandry practices or aspects of the human–animal relationship.

Nevertheless, in multispecies contexts, the simultaneous coexistence of dogs and cats may alter management choices and relationship dynamics, as well as the perception of the animals’ emotional and social capabilities [28].

Therefore, the aim of this study was (1) to investigate possible differences in the management of dogs and cats in multispecies households, (2) to identify management patterns in multispecies owners, as well as human- and animal-related factors associated with them, and (3) to assess the possible link between the owners’ management choices and their perception of the relationship with their dogs and cats.

## 2. Materials and Methods

All participants had to provide their written consent to participate in the study. No sensitive information concerning the participants’ identity or health was collected through the questionnaire. The focus of the investigation was on the animals, but none of them have been used for research purposes. Animals have not been treated in any way or submitted to any experimental protocol. Thus, ethical approval was not required. Different data collected with the same questionnaire have already been published in peer-reviewed journals [10,29].

### 2.1. Participants

This questionnaire-based study targeted individuals who owned both dog(s) and cat(s). Data were collected over a three-month period (from May to July 2014) from 1270 Italian residents who voluntarily and anonymously participated in the survey.

The relative response rate could not be calculated because the questionnaire was distributed via email and social networks.

### 2.2. Procedure

The study was part of the broader “RandAgiamo^®^” project, which aimed to reduce pet abandonment and increase the adoptability of shelter dogs in the Umbria region, in the center of Italy for details, see Menchetti et al. [30].

The questionnaire was distributed online sharing a web link via emails to acquaintances of the authors and posting it on main social networks. In order to increase the number of respondents, a virtual snowball sampling technique was used, where participants were asked to forward the email to their contacts or share the post on their profile page. The questionnaire began with an introduction explaining the purpose of the study, the prerequisites for participating in the survey (being 18 years or older and currently owning at least one dog and one cat), and instructions on how to complete the survey. In case the respondents had more than one dog/cat, they were asked to refer to the first pet acquired still living in the household. This common rule was implemented to avoid possible biases due to the owners choosing the animals according to their personal preference, which may have influenced their response to the quality of the pet–owner relationship and the type of management. The respondents had to give informed consent to the collection and processing of personal data according to the Italian privacy law. All questions were optional.

The questionnaire was composed of 63 items divided into four sections covering a wide range of information on the participant’s demographic characteristics, dog and cat management, the perception of the dog and cat’s personality traits, the relationship between the dog and the cat, and the relationship between the pets and the owner. Some questions were selected from previous studies [4,14,22,26], and two experts added additional questions related to the research topic. The pet personality section was described in Menchetti et al. [10], while only the parts of the questionnaire related to pet management and pet–owner relationship were used for this study. The variables analyzed are presented in Table S1 and were divided into four parts, as follows.

Section A included questions concerning the participant’s demographic data (age, gender, pet expertise, and the number of pets owned).

Section B consisted of multiple-choice questions concerning their dogs’ physical characteristics (age, sex, neutering status, size, and breed) and management (age at acquisition, age at separation from the mother, age at neutering, training, and living and sleeping habits). To simplify the analysis of the association between pet–owner relationships and characteristics of owners and their pets, sleeping areas for dogs and cats were categorized into “Outdoor or enclosed space”, “Home” (including “Home area” and “Free in the home” responses), “Bedroom” (including “Bedroom” and “On the bed”), and “Other”.

Section C contained the same questions as Section B but about cats.

In Section D, the participants were asked to identify the primary caregiver of the pets and describe their relationships with both the dogs and cats using a multiple-choice question.

### 2.3. Statistical Analysis

The survey data were entered into a Microsoft Excel^®^ spreadsheet and transferred into the statistical software SPSS^®^ Statistics version 23 (IBM, SPSS Inc., Chicago, IL, USA) for analysis. The level of statistical significance for all tests was set at <0.05.

Chi-square goodness of fit tests were used to compare the observed distributions of categorical variables of owner and pet demographic characteristics with the expected probability distributions (each assuming all categories equal). The Pearson chi-square and z-test were used to evaluate the independence of demographic characteristics of dogs and cats. The McNemar or Bowker tests were used to evaluate the agreement between the types of management and relationships with the owners of dogs and cats living in the same household. Microsoft Excel^®^ 2007 was used to build polar graphics.

Only valid answers were used for analyses and data presentation of each variable while missing answers were excluded. Only questionnaires with valid responses for both species were used to make paired comparisons (McNemar and Bowker tests). The missing answers never exceeded 6%.

A Two-Step Cluster Analysis (TCA) was used to identify groups of owners with similar pet management practices (Section D; [31]). The two resulting clusters were compared to find the association with demographic variables using the chi-square (and z-test) or independent *t*-test.

## 3. Results

### 3.1. Demographic Characteristics of Owners and Pets

#### 3.1.1. Owners

The majority of participants were women (91.2%; χ^2^(1) = 862.9, *p* < 0.001). The age of the participants ranged from 18 to 70 years old, but the majority were between 26 and 40 years old (44.1%) and between 41 and 55 years old (31.5%; χ^2^(3) = 395.9, *p* < 0.001). Few participants owned more than five dogs (3.1%) or cats (14.2%), while most of them owned 1 (56.4%) or 2–5 dogs (40.5%; χ^2^(2) = 545.8; *p* < 0.001) and 1 (36.9%) or 2–5 cats (48.9%; χ^2^(2) = 225.4; *p* < 0.001). However, a higher number of participants owned only one dog, rather than one cat, and 2–5 cats or more rather than 2–5 dogs or more (χ^2^(2) = 145.4; *p* < 0.001). Table S2 shows the participants’ demographic characteristics.

#### 3.1.2. Dogs

Table 1 shows detailed demographic data of dogs and cats as well as the comparison between species. Most of the dogs were females (χ^2^(1) = 16.6; *p* < 0.001) and older than 2 years (χ^2^(3) = 579.1, *p* < 0.001). There were more spayed females than castrated males (χ^2^(1) = 218.7, *p* < 0.001). The total neutering rate of dogs was 56.7% (χ^2^(1) = 22.9, *p* < 0.001), and many of the dogs had been neutered before 3 years of age (χ^2^(2) = 439.9, *p* < 0.001). Owners of purebred dogs (45.1%) reported a lower dog neutering rate than owners of mixed breed dogs (65.7%; χ^2^(1) = 51.5; *p* < 0.001). Similarly, owners of single dogs (54.4%) reported a lower dog neutering rate compared with those owning from 2 to 5 dogs (61.6%; χ^2^(2) = 6.9; *p* < 0.05). More than half of the participants (χ^2^(10) = 3166.9; *p* < 0.001) owned a mixed-breed dog. The Golden Retriever was the most represented breed (10.3%), with other breeds also present in small percentages. The majority of the dogs were left with their mother until 3 months of age at most (χ^2^(4) = 966.4; *p* < 0.001). However, most of them were acquired before they were 3 months old (χ^2^(3) = 880.5; *p* < 0.001).

#### 3.1.3. Cats

Cats of different sexes were equally represented (χ^2^(1) = 0.3; *p* > 0.1). The vast majority of the cats were neutered (χ^2^(1) = 776.0, *p* < 0.001). There was no difference in neutering rate between males and females (χ^2^(1) = 0.2, *p* > 0.1). More than half of the cats (χ^2^(1) = 926.6, *p* < 0.001) had been neutered before 3 years of age and were older than 2 years (χ^2^(3) = 411.4, *p* < 0.001). Neutering rate was lower for cats owned by participants younger than 40 years (86.6% and 87.3% for participants aged 18–25 years and 25–40 years, respectively) than those with an age range of 41–55 years (92.5%; χ^2^(3) = 8.5; *p* < 0.05). Furthermore, it was reported to be lower by participants who owned one cat (52.7%) compared with those owning more than five cats (64.0%; χ^2^(2) = 6.7; *p* < 0.05). The gender of the respondents did not affect the cat neutering rate.

Most of the cats (χ^2^(4) = 2117.7, *p* < 0.001) were mixed breed (72.6% of them were European cats) and remained with their mother for up to 3 months (χ^2^(4) = 596.3, *p* < 0.001), although they were usually acquired before 3 months of age (χ^2^(3) = 1599.7, *p* < 0.001).

#### 3.1.4. Demographic Differences Between Dogs and Cats Living in the Same Household

Table 1 compares the demographic characteristics of dogs and cats, including statistical values and degrees of freedom. Within our sample, the percentage of cats under 2 years of age was higher than the percentage of dogs for the same age interval (*p* < 0.001). Cats and dogs living in the same household were both mixed breed in 51.6% of cases and both purebred in only 5.4% of cases. However, purebred dogs were more represented than purebred cats (*p* < 0.001), and 40.0% of the participants owned a purebred dog and a mixed-breed cat (*p* < 0.001). Female dogs were represented more than female cats (*p* < 0.05). The neutering rate was higher in cats than in dogs (*p* < 0.001). More than half of the participants (52.9%) had neutered both the cat and the dog, whereas 36.1% had neutered their cat but not their dog (*p* < 0.001). More cats were neutered before 3 years of age (*p* < 0.001) and were acquired before 3 months of age (*p* < 0.001) compared to dogs. The number of cats left with their mother for less than 1 month was higher compared to dogs (*p* < 0.001).

### 3.2. Management of Dogs and Cats Living in the Same Household

#### 3.2.1. Living and Sleeping Habits

**Figure 1:**
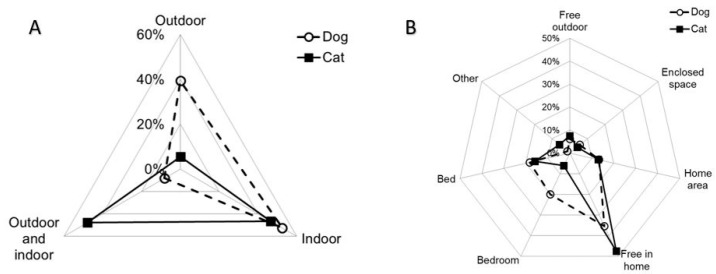
Radar graphics representing diurnal living (**A**) and sleeping (**B**) habits of dogs and cats. Each point shows the proportion of dogs (empty circle) and cats (square black) kept in a given location.

Most of the dogs lived only indoors or only outdoors (χ^2^(2) = 393.8, *p* < 0.001; Figure 1A) while most of the cats lived both inside and outside the house or only inside (χ^2^(2) = 441.8, *p* < 0.001; Figure 1A). We found agreement in living habits between dogs and cats living in the same household only in 19.4% of cases (Bowker test: *p* < 0.001; Table S3).

Most of the dogs were free to sleep in the home, in the bedroom or on the bed (χ^2^(6) = 714.2, *p* < 0.001; Figure 1B). About half of the cats slept free in the home (χ^2^(6) = 1568.5, *p* < 0.001; Figure 1B). Although the statistical test suggested disagreement (Bowker test: *p* < 0.001; Table S4), we found concordance in sleeping habits between dogs and cats in almost half of the owners (43.3%).

#### 3.2.2. Person Caring for the Dogs and Cats

Only two participants (0.2%) indicated that a non-family member managed their pets (two cats). For this reason, these answers were excluded from the statistical analysis. We found an agreement on the person managing the dog and cat living in the same household (*p* = 0.183): sixty-eight percent of the participants managed their dog and cat themselves, 13.1% managed their dog but not their cat, 11.1% managed their cat but not their dog, while in 7.7% of cases, another family member managed both the animals.

#### 3.2.3. Cluster Analysis of Pet Management

**Figure 2:**
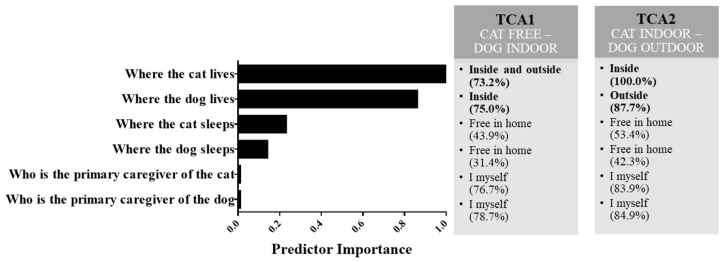
Cluster analysis of pet management. The left-side panel shows the variables included in the cluster analysis and their importance in differentiating the clusters. The questions that most contribute to cluster formation were related to the living habits of pets. The right panel describes the two clusters including the relative contribution of the categories to the overall cluster model. In TCA1 (named “Cat free-Dog indoor”), owners keeping their cat both indoors and outdoors and their dog indoors prevailed (in bold), while in TCA2 (named “Cat indoor-Dog outdoor”), owners keeping their cat indoors and their dog outdoors prevailed (in bold). The other variables did not contribute to the clustering solution as the prevailing categories coincided in the two clusters.

Cluster analysis was carried out with 1218 participants, using the items related to pets’ living habits, pets’ sleeping habits, and owners’ role as primary caregivers. The items included in the TCA are listed in Figure 2. From the analysis, two clusters emerged, with a larger sample size in cluster 1 (TCA1; 65.3% of participants) than in cluster 2 (TCA2; 34.7% of participants). Goodness of fit was achieved, with a fair average Silhouette Coefficient equal to 0.30. The most important predictors of clusters were “Where the cat lives” and “Where the dog lives” (Figure 2). In TCA1 (named “Cat free-Dog indoor”), owners who kept their cat both indoors and outdoors and their dog indoors prevailed; in TCA2 (named “Cat indoor-Dog outdoor”) owners who kept their cat indoors and their dog outdoors prevailed (Figure 2). The other variables did not contribute to the clustering solution since “Cat sleeps free in the home”, “Dog sleeps free in the home”, “Me, I take care of my cat”, and “Me, I take care of my dog” were the most frequent categories both in TCA1 and TCA2 (Figure 2).

#### 3.2.4. Association Between Clusters of Management and Demographic Characteristics of Owners and Pets

There was no association between participants’ gender and clusters (Table 2). Management was associated with the age of the respondents and the number of pets they owned. Participants under 25 years of age were more represented in the “Cat free-Dog indoor” (TCA1) than in the “Cat indoor-Dog outdoor” cluster (TCA2; *p* < 0.05). Comparing clusters, a higher proportion of the members included in the cluster “Cat indoor-Dog outdoor” had only one dog (*p* < 0.001) or one cat (*p* < 0.001).

With regard to the relationship between management and pet characteristics (Table 3), the dog size (*p* < 0.001), cat neutering (*p* < 0.05), and cat breed (*p* < 0.01) significantly differed between management clusters. Specifically, the proportion of owners who had a neutered cat, a purebred cat and a small dog was higher in the “Cat indoor-Dog outdoor” cluster. Contrastingly, intact cats, mix-breed cats, and large dogs were more frequently observed in the “Cat free-Dog indoor” cluster.

### 3.3. Pet–Owner Relationship

#### 3.3.1. Owner’s Perception of the Relationship with Cats and Dogs

Despite the significance of the Bowker test (*p* < 0.001), more than half of the participants (73.1%) described their bond with their dog and with their cat in the same terms: 61.1% as ”loving”, 11.2% as “friendly”, 0.4% as “only based on caregiving”, 0.2% as “indifferent”, 0.2% as “conflictual” (Table S5).

#### 3.3.2. Association Between Perceived Pet–Owner Relationship and Management of Pets

Since nearly all respondents identified their relationship with their pets as either friendly or loving (97% and 93% for dogs and cats, respectively), the analysis only evaluated the differences in management practices between these two types of relationships.

**Figure 3:**
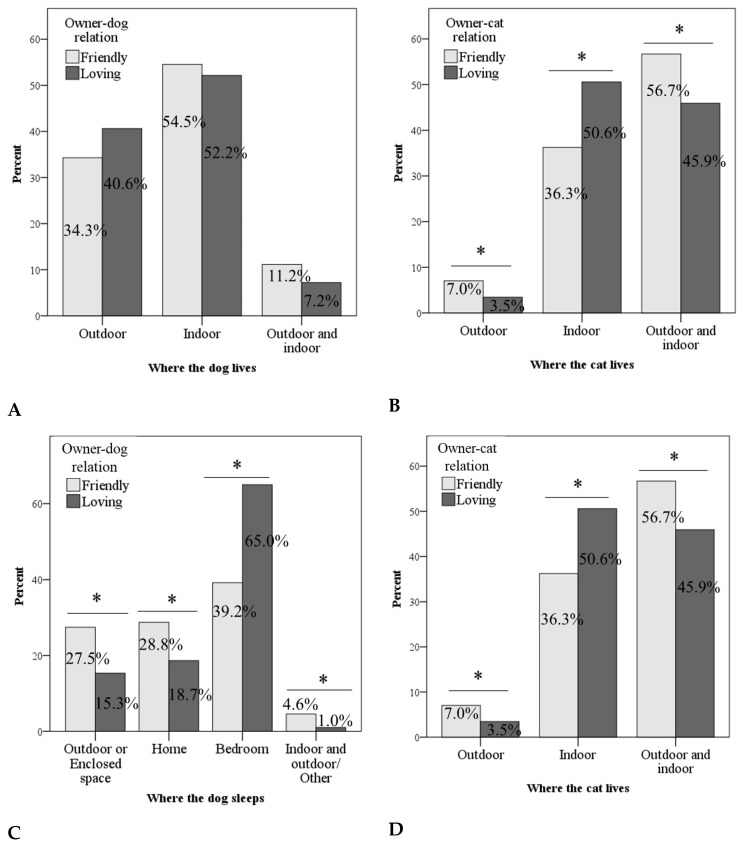
Associations between owner–pet relationship and living (**A**,**B**) and sleeping (**C**,**D**) habits of pets. * *p* < 0.05.

The dog–owner relationship was not associated with the place where the dog lives (χ^2^(2) = 5.9, *p* = 0.053; Figure 3A); instead, it was associated with the place where the dog sleeps (χ^2^(3) = 38.6, *p* < 0.001; Figure 3C). Moreover, we found associations between cat–owner relationship and the place where the cat lives (χ^2^(2) = 20.6, *p* < 0.001; Figure 3B) and sleeps (χ^2^(3) = 24.0, *p* < 0.001; Figure 3D). Specifically, a higher proportion of owners describing their relationship with their pet as loving kept their cat indoors (50.6%; Figure 3B) and allowed their dog (65.0%; Figure 3C) and cat (50.1%; Figure 3D) to sleep in the bedroom compared with those who described the relationship with their pets as friendly.

## 4. Discussion

This study aimed to explore possible differences in pet owners’ management practices for dogs and cats living in the same household and whether these practices were associated with the participants’ perception of their relationship with their pets.

### 4.1. Dog and Cat Demographic Differences and Implications for Management and Welfare

Dog and cat demographic differences observed in this study suggest that multispecies owners may manage their pets differently depending on the species. First of all, most of the respondents owned only one dog but two or more cats, supporting previous data from the US, where the average number of cats owned per household was greater than that of dogs (1.78 cats versus 1.46 dogs [32]). This is an expected finding since previous studies suggest that people perceive cats as less physically and emotionally demanding, as well as less expensive than dogs [11,26].

The neutering rate was 57% for dogs and 89% for cats. For both species, it was higher than that reported in previous studies conducted in Italy (dogs: 20–40% [33,34,35]; cats: 45–78% [22,34,36]). Percentage differences may be due to the fact that, contrary to previous research, this study focused only on multispecies households. Since pet owners often believe neutering to be a panacea intervention to reduce the display of a wide range of undesirable behaviors in their companion animals [37], this intervention may be implemented to further facilitate interspecies cohabitation in multispecies households.

Conversely, the overall higher percentage of neutered cats compared to dogs reflects findings from previous studies not focused on multispecies households [11,38,39,40]. One possible explanation for interspecies difference is that neutering relates to breed, which may, therefore, act as a confounding factor in the association between neutering and species. In our study, the number of purebred dogs was more than five times that of purebred cats. Previous studies suggest that owners of purebred animals are less likely to have them neutered [41], either because they want the animals to reproduce [39,41] or because they do not have to comply with any mandatory requirement to neuter by rescue organizations and shelters, as adopters of crossbred animals often do [41]. Another possible explanation is that cats are perceived as less controllable than dogs, especially if they are allowed to roam outside the house. With such management, intact cats would also be perceived as more likely to deliver unwanted kittens, perpetuate the phenomenon of stray animals, and/or engage in dangerous sexually driven behaviors (e.g., wandering, fighting, etc.) [11,39]. However, the higher neutering rate in indoor-only cats compared to cats that were allowed outdoors, as well as the higher prevalence of purebred cats confined indoors, in our study, suggests that breed and controllability may not be the sole factors to explain multispecies owners’ decision to have their cats neutered more frequently than their dogs. The believed and actual effects of neutering on dog and cat undesirable behaviors and the consequent impact on the owner’s quality of life [34,42] and the scientific and public knowledge on possible collateral effects of neutering [39,43,44], as well as the costs of the intervention [45], differ between the two species and may all contribute to influence neutering-related decision making. For instance, one of the main reasons why cat owners support gonadectomy is its strong effect on urine house-soiling, which may be a common expression of emotional discomfort in this species [46]. Although most cats seem to have an amicable relationship with a cohabitant dog, a good percentage of them are reported to be uncomfortable around the canine in the household or to have initiated conflictual interactions [26,27]. Therefore, urine house-soiling may be more frequently displayed in multispecies households, and so neutering may be perceived as even more beneficial. However, a recent study by Barcelos et al. [47] found no effect of the presence of household dogs on the probability of cats displaying periuria. Certainly, factors that determine species-specific differences in neutering-related decision making require further investigation. Multispecies owners may provide an optimal target for future research on this topic.

Overall, the age at neutering was lower for cats than dogs. This result seems to align with the findings reported by previous studies investigating age at neutering in dogs and cats [41,48,49]; hence, it does not seem to be unique to multispecies contexts. While some owners may actively request to neuter their cat before sexual maturity to avoid unwanted litters (dogs’ sexual behavior is often more controllable than cats’, especially in multipet households or when cats are free to roam), some others may be guided to this practice by external forces. For instance, many cat breeders still have their kittens neutered before they are sold at around three months of age for lineage maintenance purposes [50], and many veterinarians still support and/or practice early-age neutering in cats [51,52,53]. As for dogs, although the recommended neutering age may vary depending on a variety of factors (e.g., breed, size, sex, context, etc.), prepubertal interventions are usually not recommended by the scientific community [54,55,56,57]. The current species-specific discrepancy in recommended neutering age may be due to the overall fewer number of studies on the effects of early neutering on cat psychophysical welfare, as well as the fewer number of findings correlating early neutering to behavioral and physical alterations in this species [58], compared to their canine counterpart. Furthermore, other factors, such as the belief that it may help reduce the stray cat population, which is usually larger than dogs, may affect neutering-related decision-making [43].

In this study, more cats than dogs were acquired before 3 months of age and were left with the mother for no longer than 1 month. These findings may have serious welfare implications [59]. Just like for dog puppies [60], maternal caregiving behavior, as well as socialization with conspecifics during the sensitive periods, which in cats are between 2 and 8 weeks, is fundamental for the appropriate psychological and emotional development of kittens [59,61,62]. In fact, previous findings suggest that kittens separated from the mother before or at 8 weeks are more likely to display aggression and stereotypic behaviors compared to kittens separated at 12 weeks [63,64]. Previous literature does not seem to clarify whether there is a pattern for earlier acquisition among cat owners compared to dog owners. More research is necessary to determine whether such a trend may be unique to or more pronounced in multispecies contexts.

Collectively, all the above-mentioned differences in dog and cat management practices seem to delineate a reality in which cat welfare is somewhat overlooked compared to dog welfare. In such context, pet owners’ decisions are likely to represent only a small piece of a much more complex puzzle that also involves a lack of control over stray population, abandonment, and inappropriate rescue interventions and adoptions. However, greater attention towards the welfare of dogs compared with that of cats is not so surprising since multispecies owners (i.e., of both dogs and cats) tend to attribute far greater emotional capabilities to their canines rather than their feline companions [28].

### 4.2. Patterns of Pet Management (Indoor vs. Outdoor) and Associated Factors

The most represented living habits for the pets in our sample were “indoors, only” or “outdoors, only” for dogs, and “indoors, only” or “free to roam outdoors” for cats. Overall, living habits for dogs and cats within the same household were the same only in 19% of cases. These species-related differences are expected as they most likely relate to the limitations dictated by the type of building the animals live in. For instance, it would be impossible for owners living in houses without gardens—or even with gardens, for safety reasons—to allow their dogs to freely go in and out of an open door, while cats may still do it through windows or cat flaps. Furthermore, while dogs living “outdoors only” in urban or suburban areas are likely kept in fenced yards and/or under the owner´s responsibility at all times, cats with the same living habits are often considered strays rather than pets, even when cared for by specific people. Conversely, sleeping habits for dogs and cats in the same household matched in almost half of the cases, with 82.5% of cats and 86% of dogs reported sleeping inside the house. Moreover, in more than two-thirds of the cases, the participants identified themselves as the person taking care of both the dog and the cat. This suggests that the level of commitment of our respondents towards both their cats and dogs was fairly high.

Interestingly, among the variables included in the cluster analysis (pet living habits, pet sleeping habits, and person caring for the pet), only dog and cat living habits were identified as good predictors of two distinctive pet management patterns across our pet owner sample. Specifically, we found that one management pattern—which comprised a higher number of respondents—was characterized by keeping the dog indoors and the cat free to roam outdoors and indoors, whereas the other one was characterized by keeping the dog outdoors and the cat only indoors. These clusters may represent husbandry practices that reflect the level of owners´ attention to the ethological needs of their pets. Although allowing cats to free-roam is not a risk-free decision [65], the negative psycho-emotional consequences of keeping cats confined are well described in previous studies [20,66,67,68]. For instance, indoor-only cats are more likely to suffer from behavioral and stress-related disorders [20,69,70,71]. On the other hand, pet dogs kept outdoors may suffer from limited interactions with their human companions [72]. It must be said that outdoor living may not necessarily have negative effects on dogs, as it may depend on the type of environment, whether they are confined or their movements are restricted, as well as if other conspecifics are present to appease their social motivation. However, in our sample, the “Dog outdoors” group comprised a higher percentage of owners with only one dog than the “Dog indoors” group.

To further support the hypothesis that these clusters relate to the owner’s attention to the pet’s ethological needs, there is the finding that the “Cat free-Dog indoors” group is composed of a higher percentage of owners under 25 years of age. Multiple studies have found young people to have a more positive attitude towards animals and interest in animal welfare issues than older people [73,74,75]. This may be related to a higher age-related empathy towards non-human animals or to an increasing cross-generational awareness of animal welfare and needs [76].

In this study, purebred cats were more common in the “Cat indoors” group, confirming findings from previous research [16]. Some owners may perceive their purebred cats as less skilled than crossbreeds for outdoor life [16] or, perhaps, as more valuable in monetary terms; therefore, they may be less willing to leave them uncontrolled. The higher percentage of neutered cats in the “Cat indoors” group may be due to the perceived positive effect of gonadectomy on cat undesirable behaviors, such as house-soiling/marking [37,77], aggression [67,78], and scratching of furniture [79], for which both intact sexual status and indoor management may represent risk factors [20,67,79]. It is also possible that owners who decide to provide their cats with outdoor access for ethical reasons also have a more negative perception of the impact that neutering may have on their cat’s natural behavior. Finally, we found a higher percentage of small-sized dogs in the “Dog outdoors” and a higher percentage of large dogs in the “Dog indoors” group. This finding is counterintuitive as one would expect the opposite result as a consequence of owners perceiving large dogs to need more space and/or to require more house cleaning. Hence, we are not able to suggest any logical interpretation for this specific result.

### 4.3. Pet–Owner Relationship and Association with Management Choices

Regardless of the species, almost all of the participants described their relationship with their pets as either loving or friendly. While it is probable that most people who decide to share their life with an animal will have some positive emotional bond with them, it is also possible that, due to the method of distribution of the survey, our sample is biased towards more emotionally involved owners. Furthermore, there was no significant difference in how owners perceived their relationship with their dog and cat. Contrastingly, Gonzalez et al. [12] found that Mexican multispecies owners reported higher emotional closeness with their dogs compared to their cats. Conflicting results may be caused by cultural differences between Mexican and Italian owners, as well as the type of questions administered.

The owners’ perception of their relationship with their dog did not differ according to whether the dog was kept indoors or outdoors, although it must be noted that the difference almost reached statistical significance. Previous findings suggest that owners of yard dogs are less attached to their animals than owners of house dogs [80]. Unfortunately, the use of different tools/scales that focus on different constructs of the dog–owner relationships (e.g., attachment) makes any comparative discussion very difficult. Moreover, it is possible that the volunteer-based recruitment implemented for our study biased the sample towards overall more committed owners, mitigating possible differences in relationship quality with dogs kept outdoors and indoors. Interestingly, the owner´s perception of their relationship with their cat did differ depending on whether the latter was kept indoors or outdoors, with a higher percentage of owners of indoor cats defining their relationship as loving, which explicitly included a parental type of relationship rather than friendly. A previous study by Bouma et al. [81] found that owners who perceive their cat as a child or a best friend are less likely to allow the cat outdoors than those who perceive it as just a pet. Although we used different terms to categorize the cat–owner relationship, the findings from our study suggest a similar association between the level of the owner´s emotional involvement with the cat and the management habit to confine the cat inside the house. This association may be bidirectional. It is possible that highly emotionally involved owners, such as the ones who perceive pets as their children, tend to show higher levels of protective behavior towards their cats [81,82]. It is also possible that the higher frequency of cat–owner interactions facilitated by keeping the cat in a limited space promotes higher owner emotional attachment [19,83]. This may be particularly evident in multispecies households where cats may spend more time outdoors to avoid interactions with the household dogs. It must be pointed out that these results only reflect the owner´s perception of their relationship with their pet. These data, as with most data obtained from questionnaire-based studies, do not provide information on the pet´s perception of their relationship with their owner [19]. Furthermore, a stronger bond with a pet, as reported by owners, should not necessarily be considered a positive indicator of animal welfare [19]. For instance, higher self-reported affection and attachment of the owner towards the pet has been associated with dysfunctional pet–owner dyads [84], as well as higher owner neuroticism [84,85], which is, in turn, a risk factor for both restrictive management practices (e.g., denying outdoor access in cats) [86] and psycho-emotional disorders in companion animals [87,88]. Contrary to living habits, sleeping habits were associated with the perceived type of relationship in both dog and cat owners. Zahrin et al. [89] suggest that owners “blur the boundaries between humans and animals” and elevate their canine companions to actual family members by allowing dogs to sleep close to them. Therefore, it is not surprising that access to the bedroom at night in our study was more frequent for pets whose owners described their relationship as “loving” rather than “friendly”. Interestingly, within our sample, there were many more dogs than cats sleeping in the owner’s bedroom. Indeed, this difference may be due to the possibility of cats going out at night or their distinctive sleep–wake cycle. However, it is also possible that the presence of dogs alters the use of space by cats. For instance, dogs sleeping inside the owner’s bedroom or on the owner’s bed may prevent cats from using the same space as their resting area and/or getting physically close to the owner. Such dynamics may be quite specific to multispecies households. They should be further explored in future studies considering their potential effects on the pet–owner relationship.

### 4.4. Limitations

This study is based on a large number of respondents. However, due to its questionnaire-based nature, it may be susceptible to possible biases. Firstly, virtual snowball sampling is usually subject to gender, age, and education biases [90]. In this study, most of the respondents were women (91.2%). This is not surprising since women consistently show a higher level of participation in animal-related studies [14,91], possibly as a consequence of their more positive attitude and empathy towards animals [22,33,91,92,93]. However, the higher proportion of female participants may be partially explained by a higher proportion of female pet owners in the study area, as the National Association of Italian Veterinary Doctors (ANMVI) reported in a previous investigation [94]. Regardless of gender, a virtual snowball sampling protocol, such as the one used in this study, will likely lead to a self-selection bias [18]. In other words, the sample will consist of pet owners who are particularly committed to their animals and, therefore, willing to participate in the study [18,76]. Consistent with this scenario, approximately 70% of the owners from our study stated that they managed both their dogs and cats by themselves, and almost all the participants described the relationship with their companion animals as loving or friendly. Indeed, this type of bias reduces the heterogeneity of the responses obtained and leaves out a portion of the actual pet owner population that may collectively be characterized by different pet management habits. For this reason, our findings should not be generalized to the entire multiple-pet owner population. However, this type of methodological bias is not likely to affect the findings related to different management practices implemented on dogs and cats sharing the same household. Furthermore, online recruitment also has quite important benefits, such as being cost-effective, rapid, and able to reach a very high number of potential respondents, which may be missed if other sampling strategies were used. Nonetheless, our findings must be interpreted in light of the possible methodological biases arising from the use of a survey [33,95]. It is also important to note that all the answers referred to the first dog and cat acquired still living in the household. Therefore, we did not collect any data on the possible differences in managing multiple dogs and/or cats in the same household, which may affect certain managing practices that had a central role in our analysis, such as allowing pets to sleep on the bed or keeping pets outdoors. This may be an interesting factor to investigate in future research.

Both the location and timing of our study may represent possible limitations. As for the location, our experimental sample consisted of Italian pet owners, who likely share the same cultural background and general perception of the role of dogs and cats in human society. It is conceivable to think that the same study may yield different results if carried out in a different country or on a sample with a different cultural background. As for timing, all responses were collected in 2014 and are now 10 years old. The growing public concern for animal welfare issues and the evolving perception of the role of pets in human society [96] may be accompanied by a change in the implementation of certain management practices over the last decade. Because of the scant scientific information available on pet management practices in multispecies households, it is hard to speculate whether that is the case. Further research is needed to understand if and how pet management has evolved in multispecies contexts.

Finally, in this study, we used terms such as loving and friendly to categorize the type of owner’s relationship with the pet. However, these terms may encompass differences in strength (loving *more than* friendly), as well as in the quality of the relationship (loving *different than* friendly), which may make any conceptual analysis on the nature of the pet–owner bond more difficult. Perhaps assessing relationship dimensions of strength and quality separately may provide clearer information on the nature of pet–owner bonds, as well as on the factors that may contribute to dysfunctional relationships and associated inappropriate management.

## 5. Conclusions

According to our findings, there seems to be an overall concordance between pet management practices implemented in multispecies households and those reported by previous studies on single-species owners. Nevertheless, we found that both dog and cat neutering rates appeared to be higher than those reported in similar investigations carried out in the same geographical area. This may indicate that multispecies owners may perceive neutering as a helpful practice in promoting interspecies coexistence or facilitating overall management in multispecies contexts.

Furthermore, multispecies owners seem to apply different strategies to the management of their animals, depending on the species. Compared to dogs, some aspects of cat management seem to be less affected by the need to ensure high levels of mental and physical well-being. Such differences are likely to derive from a different perception of cats as a species rather than being peculiar to the management practices implemented by multispecies owners. However, they highlight the necessity of raising awareness on those management habits that may jeopardize the psychophysical welfare of cats. Finally, our sample of multispecies owners often had the same perception of their emotional bond with their dogs as with their cats, which usually indicated a high level of emotional closeness. In both dogs and cats, management practices that allowed for opportunities for greater sharing of routine activities were linked to higher perceived emotional closeness.

## Figures and Tables

**Table 1:** Demographic characteristics of dogs and cats and comparison between species.

Parameter				Pet		Pearson Chi-Square Test		
				Dog	Cat	χ^2^	df	*p* Value
Age								
		0–6 m		23 _a_ (1.8%)	52 _b_ (4.1%)	19.4	3	**<0.001**
		6 m^−2^ years		264 _a_ (20.8%)	315 _b_ (24.9%)			
		2–8 years		621 _a_ (49.0%)	561 _b_ (44.3%)			
		>8 years		359 _a_ (28.3%)	338 _a_ (26.7%)			
Breed								
		Mixed *		664 _a_ (54.6%)	1060 _b_ (91.8%)	413.3	1	**<0.001**
		Purebred		553 _a_ (45.4%)	95 _b_ (8.2%)			
Sex								
		Male		560 _a_ (44.3%)	622 _b_ (49.2%)	6.1	1	**0.013**
		Female		705 _a_ (55.7%)	643 _b_ (50.8%)			
Neutering rate								
		Overall		715 _a_ (56.7%)	1130 _b_ (89.1%)	335.9	1	**<0.001**
		Male		187 _a_ (33.6%)	557 _b_ (89.5%)	394.5	1	**<0.001**
		Female		528 _a_ (75.2%)	571 _b_ (88.8%)	41.5	1	**<0.001**
Age at neutering								
	Male		Before 3 years	110 _a_ (58.5%)	526 _b_ (95.1%)	161.2	2	**<0.001**
			After 3 years	62 _a_ (33.0%)	27 _b_ (4.9%)			
			Unknown	16 _a_ (8.5%)	0 _b_ (0.0%)			
	Female		Before first heat	140 _a_ (26.5%)	295 _b_ (51.8%)	143.5	3	**0.0001**
			Before 3 years	242 _a_ (45.7%)	250 _a_ (43.9%)			
			After 3 years	118 _a_ (22.3%)	25 _b_ (4.4%)			
			Unknown	29 _a_ (5.5%)	0 _b_ (0.0%)			
Age at separation from the mother								
			<1 week	31 _a_ (2.4%)	81 _b_ (6.4%)	73.2	4	**<0.001**
			Until 1 month	219 _a_ (17.2%)	344 _b_ (27.3%)			
			Until 3 months	572 _a_ (45.0%)	428 _b_ (34.0%)			
			>3 months	10 _a_ (0.8%)	7 _a_ (0.6%)			
			Unknown	438 _a_ (34.5%)	398 _a_ (31.6%)			
Age at acquisition								
			1–3 ms	736 _a_ (58.7%)	900 _b_ (72.7%)	73.8	3	**<0.001**
			4 ms^−1^ year	309 _a_ (24.7%)	247 _b_ (20.0%)			
			1–8 years	173 _a_ (13.8%)	85 _b_ (6.9%)			
			>8 years	35 _a_ (2.8%)	6 _b_ (0.5%)			
Dog size ^#^								
			Small (<10 kg)	243 (34.5%)	-	-	-	-
			Medium (10–20 kg)	250 (35.5%)	-			
			Large (>20 kg)	212 (30.1%)	-			

* Including European cats. ^#^ Not required in the cat section. Values of χ^2^, degrees of freedom (df) and *p* values (in bold when <0.05) refer to the comparison between dogs and cats (contingency table Chi-square test), while each subscript letter denotes a subset of species whose column proportions do not differ significantly from each other (0.05 level, *z*-test). The prevailing categories within each species are indicated in italics (chi-square goodness of fit tests).

**Table 2:** Associations between Two-Step Cluster group memberships related to pet management and demographic characteristics of the owners.

Demographic Characteristics of the Owners		Cluster		Pearson Chi-Square Test		
		TCA1–Cat Free–Dog Indoor	TCA2–Cat Indoor–Dog Outdoor	χ^2^	df	*p* Value
Gender of the respondent						
	Male	80 (10.1%)	29 (6.9%)	3.5	1	0.060
	Female	713 (89.9%)	394 (93.1%)			
Age of the respondent (years)						
	18–25	159 _a_ (20.0%)	53 _b_ (12.5%)	11.4	3	**0.010**
	26–40	344 _a_ (43.3%)	192 _a_ (45.4%)			
	41–55	235 _a_ (29.6%)	146 _a_ (34.5%)			
	56–70	56 _a_ (7.1%)	32 _a_ (7.6%)			
Experience						
	No experience	162 (20.6%)	77 (18.5%)	1.8	3	0.621
	Dog expert	78 (9.9%)	38 (9.1%)			
	Cat expert	40 (5.1%)	18 (4.3%)			
	Dogs and Cat expert	505 (64.3%)	284 (68.1%)			
No. of dogs						
	1 dog	395 _a_ (51.8%)	268 _b_ (66.3%)	26.9	2	**<0.001**
	2–5 dogs	337 _a_ (44.2%)	132 _b_ (32.7%)			
	>5 dogs	31 _a_ (4.1%)	4 _b_ (1.0%)			
No. of cats						
	1 cat	260 _a_ (34.3%)	171 _b_ (42.1%)	15.6	2	**<0.001**
	2–5 cats	370 _a_ (48.8%)	198 _a_ (48.8%)			
	>5 cats	128 _a_ (16.9%)	37 _b_ (9.1%)			

Values of χ^2^, degrees of freedom (df) and *p* values (in bold when <0.05) refer to the comparison between the two clusters (contingency table chi-square test) while each subscript letter denotes a subset of TCA categories whose column proportions do not differ significantly from each other at the 0.05 level (z test).

**Table 3:** Associations between Two-Step Cluster group memberships related to pet management and demographic characteristics of the pets.

Pet	Demographic Characteristics of Pets		Cluster		Pearson Chi-Square Test		
			TCA1—Cat Free–Dog Indoor	TCA2—Cat Indoor–Dog Outdoor	χ^2^	df	*p* Value
DOG	Age						
		0–6 m	14 (1.8%)	9 (2.1%)	3.9	3	0.276
		6 m^−2^ years	163 (20.6%)	88 (20.8%)			
		2–8 years	403 (50.9%)	193 (45.6%)			
		>8 years	212 (26.8%)	133 (31.4%)			
	Sex						
		Male	357 (45.1%)	182 (43.1%)	0.4	1	0.503
		Female	434 (54.9%)	240 (56.9%)			
	Neutering						
		No	357 (45.2%)	172 (41.0%)	2.1	1	0.152
		Yes	432 (54.8%)	248 (59.0%)			
	Size						
		Small	236 _a_ (29.8%)	161 _b_ (38.1%)	19.3	2	**<0.001**
		Medium	283 _a_ (35.7%)	166 _a_ (39.2%)			
		Large	273 _a_ (34.5%)	96 _b_ (22.7%)			
	Breed						
		Pure	353 (46.3%)	173 (42.8%)	1.3	1	0.261
		Mixed	410 (53.7%)	231 (57.2%)			
CAT	Age						
		0–6 m	30 (3.8%)	18 (4.3%)	5.5	3	0.139
		6 m^−2^ years	213 (26.9%)	90 (21.3%)			
		2–8 years	337 (42.6%)	203 (48.0%)			
		>4 years	212 (26.8%)	112 (26.5%)			
	Sex						
		Male	372 (47.0%)	218 (51.7%)	2.4	1	0.120
		Female	420 (53.0%)	204 (48.3%)			
	Neutering						
		No	95 _a_ (12.0%)	35 _b_ (8.3%)	3.9	1	**0.047**
		Yes	699 _a_ (88.0%)	388 _b_ (91.7%)			
	Breed						
		Mixed *	666 _a_ (93.7%)	347 _b_ (87.6%)	12.0	1	**<0.001**
		Purebred	45 _a_ (6.3%)	49 _b_ (12.4%)			

* Including European cats. Values of χ^2^, degrees of freedom (df) and *p* values (in bold when <0.05) refer to the comparison between the two clusters (contingency table Chi-square test), while each subscript letter denotes a subset of Two-Step Cluster Number (TCA) categories whose column proportions do not differ significantly from each other at the 0.05 level (z test).

## Data Availability

Data are available from the corresponding author upon request.

## Supplementary Materials

The following supporting information can be downloaded at https://www.mdpi.com/article/10.3390/ani14233465/s1. Table S1: Sections of the questionnaire investigated in this study (Sections B, C, D); Table S2: Demographic characteristics of the participants; Table S3: Agreement in living habits between dogs and cats living in the same household. Number and percentage in brackets within the total category; Table S4: Agreement in sleeping habits between dog and cat living in the same household. Number and percentage in brackets within the total category; Table S5: Agreement in the owners’ perception of their relationship with their dog and cat. Number and percentage in brackets within the total category.
