# Supplementary material for: Exploring Dog and Cat Management Practices in Multispecies Households and Their Association with the Pet-Owner Relationship
Source: Animals (Basel). 2024 Nov 30;14(23):3465. doi: 10.3390/ani14233465 (PMC11639825; doi:10.3390/ani14233465)
Supplement: Supplementary file 1 [file animals-14-03465-s001.zip › animals-3268648-supplementary.pdf]

Supplementary Materials

Exploring Dog and Cat Management Practices in Multispecies Households and Their Association with the Pet-Owner Relationship

Laura Menchetti, Giacomo Riggio, Silvia Calipari, Chiara Mariti, David J. Menor-Campos and Silvana Diverio

Table S1. Sections of the questionnaire investigated in this study (Sections A, B, C, D)

SECTION A: PARTICIPANT'S DEMOGRAPHIC DATA

1. **Gender**
   - □ Male
   - □ Female
2. **Age (class in years)**
   - □ 18-25 years
   - □ 26-40 years
   - □ 41-55 years
   - □ 56-70 years
3. **Experience**
   - □ No experience
   - □ Dogs' expert
   - □ Cats' expert
   - □ Dogs and cats' expert
4. **Number of currently owned dogs**
   - □ 1 dog
   - □ 2-5 dogs
   - □ >5 dogs
5. **Number of currently owned cats**
   - □ 1 cat
   - □ 2-5 cats
   - □ >5 cats
6. **Number of currently owned pets other than dogs and cats**

(continued)

SECTION B: PHYSICAL CHARACTERISTICS AND MANAGEMENT OF THE DOG

7. **Age**
   - □ 0-6 months
   - □ 6 months-2 years
   - □ 2-8 years
   - □ > 4 years

8. **Size (only for the dog)**
   - □ Small
   - □ Medium
   - □ Large

9. **Breed**
   - □ Mixed
   - □ Purebred …………

10. **Sex**
    - □ Male
    - □ Female

11. **Neutering**
    - □ Yes
    - □ No

12. **Age at neutering**

**Male**	□ Before 3 years<br>□ After 3 years<br>□ Unknown
**Female**	□ Before the first heat<br>□ Before 3 years<br>□ After 3 years<br>□ Unknown

13. **Age at separation from the mother**
    - □ < 1 week
    - □ Until 1 month
    - □ Until 3 months
    - □ > 3 months
    - □ Unknown

(continued)

14. Age at acquisition

- □ 1-3 months
- □ 4 months-1 year
- □ 1-8 years
- □ > 8 years

15. Who trained the dog? (only for the dog)

- □ None
- □ Me
- □ Me and a trainer
- □ Other

16. Where does your dog live?

- □ Indoor
- □ Outdoor
- □ Outdoor and indoor

17. Where does the dog sleep?

- □ Enclosed space (kennel, box, terrace or garage)
- □ Home area
- □ Free in the home
- □ Bedroom
- □ On the bed
- □ Free outdoors
- □ Other

SECTION B: PHYSICAL CHARACTERISTICS AND MANAGEMENT OF THE CAT

[same questions as Section B]

(continued)

SECTION C: OWNER-PET AND PET-OWNER RELATIONSHIP

27. **Who is the primary caregiver of the dog?**
    - □ Me
    - □ A family member
    - □ A non-family member

28. **Who is the primary caregiver of the cat?**
    - □ Me
    - □ A family member
    - □ A non-family member

29. **How would you describe your relationship with your dog?**
    - □ Indifferent
    - □ Conflictual
    - □ Based on caregiving
    - □ Friendly (and playful)
    - □ Loving (affectionate and parental)

30. **How would you describe your relationship with your cat?**
    - □ Indifferent
    - □ Conflictual
    - □ Based on caregiving
    - □ Friendly (and playful)
    - □ Loving (affectionate and parental)

Table S2. Demographic characteristics of the participants

		Number and percentage
**Gender**	**Male**	111 (8.8%)
	**Female**	1157 (91.2%)
**Age of respondent (class in years)**	**18-25**	217 (17.1%)
	**26-40**	559 (44.1%)
	**41-55**	399 (31.5%)
	**56-70**	93 (7.3%)
**Expert**	**No experience**	249 (19.9%)
	**Dogs' expert**	119 (9.5%)
	**Cats' expert**	62 (5.0%)
	**Dogs and cats' expert**	822 (65.7%)
**N° dogs**	**1 dog**	686 (56.4%)
	**2-5 dogs**	492 (40.5%)
	**>5 dogs**	38 (3.1%)
**N° cats**	**1 cat**	445 (36.8%)
	**2-5 cats**	592 (49.0%)
	**>5 cats**	172 (14.2%)
**N° other animals (mean±SE)**		3±0

Table S3. Agreement in living habits between dogs and cats living in the same household. Number and total percentage in brackets.

Living habits						
		CAT			**Total**	**P value**
		**Outdoors**	**Indoors**	**Outdoors and indoors**		
	**Outdoors**	9 (0.7%)	384 (30.5%)	103 (8.2%)	496 (39.3%)	
DOG	**Indoors**	31 (2.5%)	183 (14.5%)	448 (35.5%)	662 (52.5%)	<0.001
	**Outdoors and indoors**	29 (2.3%)	21 (1.7%)	53 (4.2%)	103 (8.2%)	
**Total**		69 (5.5%)	588 (46.6%)	604 (47.9%)	1261 (100%)	

P value from Bowker test ($\chi^2(3)= 730.5$)

Table S4. Agreement in sleeping habits between dog and cat living in the same household. Number and total percentage in brackets.

		Sleeping habits								
		CAT								
		**Free outdoors**	**Enclosed space**	**Home area**	**Free in the home**	**Bedroom**	**On the bed**	**Other**	**Total**	**P value**
DOG	**Free outdoors**	30 (2.4%)	3 (0.2%)	9 (0.7%)	22 (1.7%)	2 (0.2%)	5 (0.4%)	6 (0.5%)	77 (6.1%)	<0.001
	**Enclosed space**	7 (0.6%)	22 (1.7%)	7 (0.6%)	19 (1.5%)	3 (0.2%)	9 (0.7%)	5 (0.4%)	72 (5.7%)	
	**Home area**	14 (1.1%)	7 (0.6%)	59 (4.7%)	56 (4.5 %)	6 (0.5%)	17 (1.4%)	8 (0.6%)	167 (13.3%)	
	**Free in the home**	21 (1.7%)	4 (0.3%)	32 (2.5%)	309 (24.6%)	13 (1.0%)	40 (3.2%)	25 (2.0%)	444 (35.3%)	
	**Bedroom**	10 (0.8%)	10 (0.8%)	33 (2.6%)	105 (8.3%)	39 (3.1%)	43 (3.4%)	11 (0.9%)	251 (20.0%)	
	**On the bed**	10 (0.8%)	6 (0.5%)	23 (1.8%)	80 (6.4%)	13 (1.0%)	86 (6.8%)	13 (1.0%)	231 (18.4%)	
	**Other**	2 (0.2%)	1 (0.1%)	0 (0.0%)	5 (0.4%)	1 (0.1%)	1 (0.1%)	6 (0.5%)	16 (1.3%)	
**Total**		94 (7.5%)	53 (4.2%)	163 (13.0%)	596 (47.4%)	77 (6.1%)	201 (16.0%)	74 (5.9%)	1258 (100%)	

P value from Bowker test ($\chi^2(21)$= 195.8)

Table S5. Agreement in the owners' perception of their relationship with their dog and cat. Number and total percentage in brackets.

		Pet-owner relationship						
		CAT						
**Item**		**Indifference**	**Conflictual**	**Based on caregiving**	**Friendly**	**Loving**	**Tot**	**P value**
	**Indifference**	3 (0.2%)	0 (0.0%)	0 (0.0%)	0 (0.0%)	1 (0.1%)	4 (0.3%)	
	**Conflictual**	0 (0.0%)	3 (0.2%)	0 (0.0%)	1 (0.1%)	5 (0.4%)	9 (0.7%)	
**DOG**	**Based on caregiving**	2 (0.2%)	0 (0.0%)	5 (0.4%)	1 (0.1%)	14 (1.2%)	22 (1.8%)	0.001
	**Friendly**	2 (0.2%)	3 (0.2%)	17 (1.4%)	135 (11.2%)	83 (6.9%)	240 (20.0%)	
	**Loving**	2 (0.2%)	10 (0.8%)	33 (2.7%)	147 (12.2%)	734 (61.1%)	926 (77.1%)	
**Tot**		9 (0.7%)	16 (1.3%)	55 (4.6%)	284 (23.6%)	837 (69.7%)	1201 (100%)	
